# American Association of Obstetricians and Gynecologists at Syracuse

**Published:** 1910-10

**Authors:** 


BUFFALO MEDICAL JOURNAL

A Monthly Review of Medicine and Surgery

*EDITOR*

WILLIAM WARREN POTTER, M. D.

All communications, whether of a literary or business nature, books for review and xchanges, should be addressed to the editor, 238 DELAWARE AVENUE, BUFFALO, N.Y.

VOL. LXVI. OCTOBER, 1910. No. 3

### American Association of Obstetricians and Gynecologists at Syracuse

THIS famous association held its twenty-third annual meeting at Syracuse, September 20-22, 1910, under most favorable auspices. The weather was fine, the place of meeting,—the new Hotel Onondaga,—was of the best, and the medical profession of Syracuse and the adjacent territory came in force to listen to the proceedings, manifesting enjoyment for the entire three days.

The association was most fortunate in the selection of its president, Dr. Aaron B. Miller, of Syracuse, who proved a skilful leader as well as a presiding officer of tact and forcefulness. He was supported by two vice-presidents, Dr. Charles N. Smith, of Toledo, and Dr. Raleigh R. Huggins, of Pittsburg, who were capable and active men, and who contributed in no small degree to the success of the meeting. The program was large and of a character that elicited extended and active debate, taxing the judgment and parliamentary knowledge of the presiding officers many times, but they were equal to the task. No time was wasted, while, on the other hand, the debates were not cut short when important, by the arbitrary use of the gavel. From the morning of the first day until the afternoon of the last, the interest did not lag, and at the end everybody seemed to regret that the meeting must close.

The efficient work of the committee of arrangements deserves notice. The chairman, Dr. Albert E. Larkin, together with his associates Drs. George B. Broad, Dr. Frederick Flaherty, and Dr. A. S. Hotaling, besides making ample preparation beforehand, were everywhere present during the meeting assisting the officers in making the details complete and to run without friction. The luncheon, on Wednesday, at the Onondaga Golf and Country Club, given by the medical profession of Onondaga, under the direction of this committee, was a most delightful function, complete in every way. The drive to the club in motor cars, supplied by the Syracuse doctors, was a refreshing prelude in the brisk afternoon air to an appetising menu set forth at the club.

The banquet! what shall be said of it? How shall it be described with adequate justice? It was served at The Onondaga, Wednesday evening, and proved a fitting conclusion to the social features of the meeting.

The menu card prepared with great good taste, was a booklet of eight pages, printed on Japanese paper with deckled edges and silken fly leaves:

THE MENU.

"O, hour of all hours, most blest upon earth:
Blessed hour of our dinners. . .
. . . . . . . . .
Never, never, ah, never, earth's luckiest sinner
Hath unpunished forgotten the hour of his dinner."
—Meredith.

MATERIAL

"Go to then, and feast thee to thy heart's content."
—Shakespeare.

Frivolites

Blue Points

Consommé Double Rossini

Celery Salted Almonds Olives

Darne de Saumon d'Orleans

Noisettes d'Agneau de Pauillac Judic
Pomme Ninon

Asperges d'Argenteuil, Sauce Vierge

Sorbet au Louis Roederer

Squab en Cocotte Fritzi Scheff

Salade Caprice

Glacé Fantaisie

Petites Fours

Roquefort and Cream Cheese

Café

Martini Cocktail

Haut Sauterne

Moet & Chandon's
White Seal

Clinton Lithia

Cigars
Cigarettes

MENTAL

"Now good digestion wait on appetite and health on both."
—Shakespeare.

An Address
James Roscoe Day, S.T.D., LL.D.
Chancellor Syracuse University

French Dialect Readings
Mr. Douglas E. Petit

Entertainment
Mr. Edwin R. Weeks

"The goodness of the night on you."
—Shakespeare.

OFFICERS—president, Aaron Benjamin Miller, Syracuse; vice-presidents, Charles North Smith, Toledo; Raleigh Russell Huggins, Pittsburg; secretary, William Warren Potter, Buffalo; treasurer, Xavier Oswald Werder, Pittsburg.

BANQUET COMMITTEE—Albert Edwin Larkin, Syracuse; Frederick Flaherty, Syracuse; Albert Steuben Hotaling, Syracuse; George Burney Broad, Syracuse.

When the one hundred men sat down to the tables at eight o'clock, the Hiawatha room of The Onondaga, in which the banquet was served, presented a brilliant spectacle. Kapp's orchestra sounded the march, led by President Miller and Chancellor James R. Day, of Syracuse University, the other ninety-eight following and taking seats in their appointed places. At the president's table besides himself were seated Chancellor Day, Mr. Douglas E. Petit, of Syracuse; Dr. Herman E. Hayd, the president elect, and seven or eight ex-presidents of the association. The music, the decorations of the tables with flowers, and the general arrangements of the feast were all most appropriate, while the banqueters themselves were in excellent spirits.

Chancellor Day was introduced at about 9.30, by the president, Dr. Miller, with some pleasantry and other remarks befitting the occasion. The chancellor, despite some throat irritation, was in a happy mood and kept the listeners at attention, who with alternate laughter and applause punctuated his address. It is quite impossible to do justice to the chancellor's speech in these pages, but when he said in effect: "I respect you that you are contributing to make a woman strong that she may bear a Man;" and, again, when he asserted with all the force of his stalwart frame and stentorian voice, that "the noted specialists in obstetrics and gynecology whom he faced were engaged in the biggest business in the world," the applause burst forth with spontaneity in prolonged and appreciative thankfulness for the tribute.

Mr. Petit favored the assemblage with charming readings in French dialect, displaying a talent that was of the highest order in method and finish. He received the generous plaudits of his hearers. Mr. Edwin R. Weeks, of Binghamton, a professional entertainer, then took the floor and for more than half an hour kept his audience in roars of laughter and in rapturous applause, by his imitations, impersonations, and comicalities. His last number was a burlesque representation of Caruso in a selection from the opera of Rigoletto, *"La Donna è mobile."* It was a superb caricature both in make-up and in musical skill.

A delegation of students from the medical college of the University sang old-time college and modern popular songs before the chancellor began his speech, in which nearly all the physicians, young and old, joined with zest, including the chorus of "Yip I addy I ay," and "There will be a hot time in the old town."

The hour of midnight was reached just as the banquet broke up, with everybody in the best of good feeling and satisfaction.

The third day was one of the best last day's work the association ever did, and when the hour of adjournment came good-bys were said with regret.

The following named officers were elected: president, Dr. Herman E. Hayd, Buffalo; vice-presidents, Dr. Henry Schwarz St. Louis and Dr. Lewis C. Morris, Birmingham; secretary Dr. William Warren Potter, Buffalo; treasurer, Dr. Xavier Oswald Werder, Pittsburg; executive councillors, Dr. Aaron B. Miller, Syracuse; and Dr. John W. Keefe, Providence. The next meeting will be held at Louisville, September 26, 27, and 28, 1911.

